# Commentary: Okay, now what?

**DOI:** 10.1016/j.xjon.2021.02.004

**Published:** 2021-02-16

**Authors:** Charles B. Huddleston, Marye J. Gleva

**Affiliations:** a Division of Cardiothoracic Surgery, Department of Surgery, St Louis University School of Medicine, St Louis, Mo; b Division of Cardiology, Department of Medicine, Washington University School of Medicine, St Louis, Mo

**Figure:**
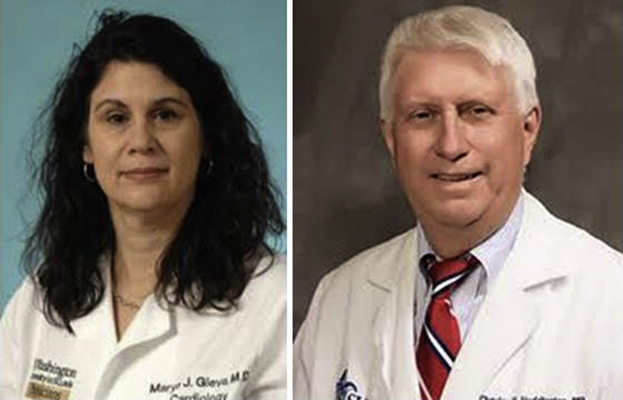
Marye J. Gleva, MD, and Charles B. Huddleston, MD

**Central Message**

Endoepicardial asynchrony in the presence of atrial extrasystoles may play an important role in post-operative atrial fibrillation. It is not clear what we can do with that bit of information.

See Article page 120.

Atrial fibrillation following cardiac surgery continues to be a vexing problem. It appears clear that the use of amiodarone as prophylaxis against postoperative atrial fibrillation (POAF) is effective although certainly has not eliminated the problem. Because the underlying etiology of POAF is complex and likely multifactorial, research getting at the basis for this is frustrating and difficult. The same could have been said about spontaneous atrial fibrillation as late as the 1980s, before the pioneering work of John Boineau, James Cox, and Richard Schuessler, who devised a computerized mapping system for the human right atrium.^1^ This led to a better understanding of atrial fibrillation and generated the multimillion-dollar surgical/medical–industrial complex devoted to the treatment of this arrhythmia.

In this issue of *JTCVS Open*, Kharbanda and colleagues^2^ from the Erasmus Medical Center in Rotterdam present their work on endoepicardial asynchrony and the impact of atrial extrasystoles on POAF. This work involved complex mapping of the endocardial and epicardial surfaces of the right atrium during cardiac surgery in adults using 256 electrodes. We applaud the efforts to conduct this very difficult study. There is also value in the discussion, which brings up the integration of the atrial electrophysiology and histology. Their findings suggest a potential mechanism for POAF. What is this mechanism? Well, it's complex and likely multifactorial but may indeed have something to do with the asynchronous conduction between the endocardial and epicardial surfaces of the atrium, particularly in the presence of atrial extrasystoles.

The authors describe this as a “pilot” study. Pilots take us from one place to another. So where do we go from here to reduce POAF? Kharbanda and his fellow investigators suggest more studies. The pursuit of answers to these questions perpetuates the innovative work of James Cox and others. At the moment, however, it is a bit difficult to tell where this is going to take us from our current position of prophylactic amiodarone for POAF.
